# The socio-ecological contradictions of land degradation and coastal agriculture in south India

**DOI:** 10.1177/25148486221079720

**Published:** 2022-03-07

**Authors:** C.M. Pratheepa, Rengalakshmi Raj, Shreya Sinha

**Affiliations:** 124709 MS Swaminathan Research Foundation, India; 124709 MS Swaminathan Research Foundation, India; 2152 University of Cambridge, UK

**Keywords:** Land degradation, coastal agriculture, shrimp aquaculture, inequality, India

## Abstract

This paper examines the drivers and impacts of degradation of agricultural land in a village in a South Indian coastal delta. Drawing on insights from political economy and political ecology scholarship, it argues that there is a dialectical relation between land degradation and social inequalities, i.e. the biophysical degeneration of land is not only caused by human and environmental factors but it also generates social contradictions which further intensify both land degradation and social inequalities. The paper demonstrates, firstly, that coastal land degradation has been generated in the form of soil salinity through a confluence of the inherent environmental vulnerabilities of the region, forces of commoditization (especially those associated with shrimp aquaculture), existing social hierarchies and state policy. Secondly, it shows that social groups differentiated by caste, class and gender have experienced these changes differently. A small, relatively well-off section of the locally dominant caste landowners has benefitted from shifting from paddy to shrimp aquaculture, which is leading to a further increase in salinisation. Others have to bear the brunt of decreasing productivity of land for crop cultivation, leading them to abandon or reduce cultivation and increase dependence on non-agricultural sources of income, especially through male outmigration. Women's burden of work has increased while their capacity to earn wage-income has declined. Without any concerted state effort to either restore the land or to alleviate the widespread agrarian distress, land degradation has developed into a continuously evolving downward spiral, but one where there will still be some winners and many losers.

## Introduction

As the space where sea and land are in ‘dynamic equilibrium’ (Bandyopadhyay et al., 2011), coastal environments are relatively ecologically fragile. They are among the most vulnerable to the effects of climate change, especially through sea-level rise, coastal flooding and extreme weather events like cyclones, across the globe. A growing body of scholarship engages with the potential impacts of such changes and adaptive strategies in coastal areas (e.g. Glavovic et al., 2019). However, the link between coastal land degradation, agriculture and social change remains a relatively neglected area of research within studies on coastal environmental degradation, even though it is a globally widespread problem (see Shahid et al., 2013).

The present paper addresses this gap in scholarship by focusing on the socio-ecological contradictions of degradation of agricultural land in a coastal delta region in south India. We know that what counts as degradation depends on the purpose of land use in consideration and the social standpoint one adopts (Blaikie and Brookfield, 1987). We defined land degradation as the incapacity of land to produce crops as a result of soil salinisation as this had implications for rural livelihoods and food security of the local population. Several studies point to the particular biophysical vulnerability of coastal deltas, especially in developing countries, to climate change (Edmonds et al., 2020; Lwasa, 2015; Tessler et al., 2015). This research, on the other hand, contributes to the relatively thinner scholarship that develops a political ecology or political economy understanding of the social and environmental processes that contribute to and sustain coastal soil salinisation (e.g. Paprocki and Cons, 2014).

It is widely acknowledged that land degradation is caused by both environmental and social factors. We build on this to argue, however, that how and why such degradation is sustained or intensified is shaped by local social relations and wider political economy. Moreover, not only does land degradation have a differentiated social impact but the latter generates conditions that sustain or intensify conditions of degradation. There is, in other words, a dialectical relation between land degradation and social inequalities.^1^ In the context of our field site, we demonstrate that biophysical specificities of the coastal environment combine with local inequalities of caste, class and gender, the policy push towards shrimp aquaculture and the state's narrow view of and inaction towards coastal environmental problems to generate land degradation as a continuously expanding process. As a result, the income and food security of landed poor and the landless households, especially the women, are most affected while the relatively wealthier landed households engaged in shrimp aquaculture may be deemed ‘winners’. This tells us that remedying land degradation requires political will to act in the interest of the most marginalised section of rural society.

This paper is divided into seven sections. This introduction is followed by a review of how land degradation has been studied in the closely aligned fields of political ecology and political economy. Our understanding of political ecology draws on Blaikie and Brookfield (1987: 17), i.e. as a field that ‘combines the concerns of ecology and a broadly defined political economy… (including) the constantly shifting dialectic between society and land-based resources, and also within classes and groups within society itself’. We argue that social relations are crucial to understand the process and impact of land degradation in any context. Moreover, rather than assuming particular types of impact on particular groups (e.g. on women or smallholders), we need to recognise social groups and individuals as embodying interests that may drive or amplify degradation. Finally, we suggest that both local and non-local factors (environmental, economic and political) shape the processes and impacts of land degradation.

Section III describes the field site and the methods of data collection. Section IV analyses the causes of land degradation while Section V describes the changes in land use in the field site, including changes in cropping patterns across seasons. Section VI presents the differentiated social impact of these changes, sub-divided by land and caste relations, labour and male outmigration, and women's work. Finally, section VII summarises the main arguments and concludes.

## Land degradation in social context

That any kind of environmental degradation, including land degradation, is closely intertwined with power-laden human-environmental relationships has been established by large numbers of studies in the related fields of political ecology and political economy, with some landmark ones in the 1980s and 1990s (Blaikie, 1985; Blaikie and Brookfield, 1987; Peet and Watts, 1996; Redclift, 1987). However, even within this broad understanding, the dynamics and causality of degradation have been widely debated.

An important conceptualisation is that environmental degradation is caused by the breakdown of traditional institutions due to the advent of modernity and ‘development’. In India, scholars such as Shiva (1988) and Gadgil and Guha (1993) have argued that harmonious relationships of local communities with the environment were disrupted by colonial and capitalist development. Similar narratives may also be found in sub-Saharan Africa, where population growth featured alongside institutional breakdown in the analyses of environmental degradation (see Fairhead and Leach, 1995). In the same vein, García-Barrio and García-Barrios (1990) argued that structural transformation in Mexico led to a breakdown of collective indigenous institutions, semi-proletarianisation and rural outmigration and thus to environmental degradation.

While this view brings necessary attention to the impact of colonial and neo-colonial forces, it has been critiqued by others for constructing a selective, romanticised version of the past and of community institutions. Fairhead and Leach (1995) argue that these narratives represent ‘stabilised assumptions’ (1031) of social scientists. They argue that any vegetation change is not necessarily degradation and may in fact represent, for example, change in the preferences of local communities based on their need or the impact of wider political and economic changes. In other words, there is ‘no baseline’ (1032) for how societies value particular environmental features and these can change over space and time (see also Blaikie, 1985). In the context of India's environmental crisis, Sinha et al. (1997) have termed such narratives as ‘new traditionalist’, i.e. they construct a narrative about Indian traditions of protecting the environment, largely by marginalised/subaltern groups, and place the blame of environmental degradation on the colonial and postcolonial states. Without absolving the colonial and postcolonial states of their responsibility, they point to evidence that establishes that deforestation predates colonialism and that ‘traditional’ farming practices were not necessarily ecologically stable. Rather, the relations between social groups and nature should be understood through ‘local conditions of domination and subordination’ (82); in other words, through social relations.

This resonates with Bernstein and Woodhouse (2001) who centre social relations in their analysis of environmental change. In response to arguments that environmental change or degradation takes different forms in different contexts, they add that ‘these problems typically take different forms *for different (types of) people in the same place(s)*’ (296; italics ours). They write that ‘…the effective study of environmental change needs to incorporate, and indeed to start from, questions of whose environments/resources, and whose livelihoods are generated from access to, use and management of them, in what ways, and with what effects (both social and environmental)’ (319). Bernstein and Woodhouse suggest that the lens of commoditization and social differentiation allow us to evaluate environmental changes better as it allows us to account for the intensification of resource use independently or in response to demographic growth. An excellent example of this comes from Niazi (2003) who argues that unequal access of land is a major driver of land degradation in Pakistan. Large landholders with easy access to irrigation schemes over-irrigate their cash crops while small landholders and tenant farmers over-exploit their land to make ends meet. The result is waterlogging and salinity. Similarly, Castellanos-Navarrete and Jansen (2015)'s work on oil palm plantation in Mexico shows that far from being torchbearers of agroecology, smallholders embraced industrial oil palm cultivation (widely accepted as environmentally damaging) as it promised better income or allowed them to avoid exploitation by richer households.

We consider the framing of environmental degradation as closely tied with unequal social relations and economic and political processes in a specific historical moment as hugely generative. Rather than dismissing the significance of traditional institutions altogether, such a lens can also allow us to understand why such institutions may have been key to a given type of environmental stability in some contexts. Not only does it do away with assumptions about an ideal environmental state, it establishes environmental change as a process that is always in motion alongside changes in society. It also suggests that environmental degradation can result in both winners and losers, resulting in new social and ecological contradictions, and lends agency to those impacted by environmental change.

Several examples demonstrate these open-ended outcomes while underlining the significance of the lens through which degradation is evaluated. In a study in highland Madagascar, Unruh et al. (2010) demonstrated how local inhabitants used the conditions of erosion caused by deforestation to intensify agricultural production. This had positive implications for ‘food security and livelihood resilience’ (362). Similarly, as the forested hills of Uttarakhand in north India saw more and more forests being closed-off by the state to prevent degradation, the local women who went to collect firewood for domestic use were branded as ‘thieves’. In some places, women used the existing institutional structure to establish control over some forests and regenerate them for their use. Equally, their alienation from the state led them to steadfastly refuse to support efforts to prevent or control fires in the state-controlled forests (Sarin, 2001).

As a corollary to the above point, how degradation might impact differentiated local communities is also, of course, contingent. Several studies demonstrate that structurally disadvantaged groups are likely to be further marginalised by degradation. But an intersectional lens is necessary to capture the nuances of different contexts. For instance, environmental degradation in rural areas has been found to particularly worsen the conditions of work and life for women by increasing their household work burdens (through difficulties collecting food, fuel wood, water) or by depleting the resources through which women earned cash (e.g. fishing or non-timber forest produce) (Cruz-Torres, 2001; Kansanga et al., 2020; Nowak, 2008; Sarin, 2001; Shiva, 1988). And yet, this is not due to essentialized properties of women but rather, as feminist political ecology informs us, due to the historically and spatially-situated lived experiences of women (and men) that create differential gender interests and knowledges vis-à-vis the environment (Jackson, 1993; Rocheleau, 1995). So, it is also the case that different women within the same context might experience such changes differently. Kansanga et al. (2020), for instance, demonstrate that in northern Ghana, degradation and appropriation of common resources caused greater distress to women from households with less land. Meanwhile, those with more female children were relatively better off as they had more family labour to collect depleting food resources such as shea and dawadawa.

Not only are impacts differentiated but they may also manifest in ways that worsen the conditions of degradation. In northwestern Mexico, for example, the undermining of subsistence livelihoods of fishing and farming led rural households to adopt strategies such as poaching shrimps during the off-season which worsened the strains on the environment and their livelihoods (Cruz-Torres, 2001). Similarly, in a study that resonates closely with ours, Paprocki and Huq (2018) argue that soil salinisation resulting from the growing shrimp aquaculture in Bangladesh's southwestern coast benefitted ‘large absentee landholders and urban-based processing factory-owners’ (2) but squeezed out smallholders and landless labourers from agriculture. This in turn worsened soil salinity in these areas.

To be sure, the approach outlined here to understand the drivers and impacts of land degradation accounts less for the fact that there are ecological constraints and vulnerabilities that may exist independently of the nature of social relations (e.g. the propensity for soil salinity in a coastal area, which we discuss below). It is important, however, to not fetishize environmental conditions and understand that whether specific vulnerabilities are realised depends on socio-economic and political conditions.

Most of the literature surveyed so far also does not explicitly account for the question of scale, which is central to political ecology analysis. There is an important debate within political ecology on the conceptualisation of scale, e.g. as socio-spatial levels/hierarchies or socially produced and on how analysis of ecological and social scales might be aligned (Rangan and Kull, 2009). It is beyond the scope of our research to intervene in this debate. However, our analysis accounts for scale in the limited sense of the social and ecological factors and processes beyond the immediate field site that are driving the process of land degradation.

### Coastal land degradation

We now situate our work in relation to other studies on coastal land degradation. As mentioned earlier, the intrinsic fragilities of coastal ecosystems are being compounded by climate change and socio-economic developments. Our research is neither about coastal environments overall nor about coastal urban environments or fishing communities. Rather, it is concerned specifically with coastal land degradation as it relates to agriculture. Even beyond climate change, a propensity towards soil and water salinity, problems with drainage, flooding by tidal water and lack of freshwater for irrigation are commonly recognised as problems for crop cultivation in coastal areas (Bandyopadhyay et al., 2011).^2^ But studies that are cognizant of how social relations inform these biophysical changes are few and far between. We, therefore, find it instructive to think through our research in relation to the wider literature on coastal environmental change and degradation.

Given that across the world, a range of different economic activities take place in coastal zones (fishing, crop farming, petroleum extraction and tourism etc.), it is not surprising that the literature on coastal environments highlights the significance of human and non-human factors in shaping environmental change. But there are hardly any studies that highlight the dynamic interplay between them to determine social and environmental outcomes. Lakshmi and Rajagopalan (2000), for example, point to the multiple pressures on coastal environments, such as population growth, urbanisation and ill-conceived developmental and urbanisation projects in the coastal villages of India. However, they conceive degradation as driven by only external agents and forces, creating socio-economic problems that then need to be fixed. Similarly, Phong et al. (2017) highlight the combined effect of climate change and ‘human activities’ like aquaculture, breach of sea dyke and faulty land use planning in causing coastal erosion in coastal Vietnam. But they do not engage with the local socio-economic dynamics generating and generated by such erosion. Gould et al.’s (2020) study on Lincolnshire UK makes an important contribution by demonstrating long-term effects of coastal flooding ‘even after flood waters recede’ (p. 1546). However, here again there is only passing mention of local economic effects. While our research builds on the insights of this research, it is distinct in underlining the *relational* nature of land degradation.

In the relatively limited scholarship on coastal land degradation and agriculture, there is however a sizable scholarship which engages entirely or partly with this issue in relation to the growth of shrimp aquaculture (Abdullah et al., 2017; Haider and Hossain, 2013; Hossain et al., 2013; Lakshmi and Rajagopalan, 2000; Pàez-Osuna, 2001; Paprocki and Cons, 2014; Paprocki and Huq, 2018). They point to the adverse impacts of shrimp aquaculture in the form of aggravating soil salinity, the abandonment of crop cultivation, the decline of allied activities such as livestock and finally, the decline of food security. These studies directly anticipate some of the findings from our research. However, while only some of them (like Paprocki and Cons, 2014) disaggregate the effects by different social groups, this is an important focus for our work. On the other hand, shrimp aquaculture is not the focus of our research but has been studied as part of the wider matrix of issues associated with land degradation.

Other studies linking coastal degradation more broadly with agriculture include those that focus entirely on biophysical and infrastructural aspects (e.g. Marfai, 2011; Nguyen et al., 2019; Shahid et al., 2013) or those that assess the impacts of seawater intrusion and/or coastal erosion on livelihoods, food security or social change but without accounting for differentiated impacts (e.g. Gopalakrishnan et al., 2019; Karlsson et al., 2015; Khanom, 2016; Magsi and Sheikh, 2017).

The discussion of scale remains muted or entirely implicit in this literature, but multiple studies point to its significance. In a comparative study of fisheries in India and Indonesia, Armitage and Johnson (2006) argue that coastal degradation resulted from a ‘coalescence’ of multiple forces of globalisation. In our view, not every factor of degradation can necessarily be seen through the lens of globalisation but the idea of coalescence allows us to conceive of degradation as caused by both proximate and more distant factors. On the other hand, in the context of the Californian coast, Stuart (2010) points to the conflicting priorities of marine conservation and intensive agriculture as a result of the interests of diverse stakeholders such as agriculturalists, corporations, non-governmental organisations, regulatory bodies etc. Her analysis too suggests the need to account for varied networks within which a coastal zone may be embedded in understandings environmental change.

Through a focus on coastal agricultural land and livelihoods, therefore, our paper fills an important gap in the literature. Moreover, it examines the entire spectrum of social relations – class (albeit through the proxy of land relations), caste and gender – in this context to examine the relative gains and losses experienced in the area.

## Site and methods

With the objective of studying land degradation in a coastal delta where agriculture constitutes an important source of livelihood, our research was conducted in a coastal village panchayat (or the village level administrative unit; henceforth CVP) in Sirkali block, Mayiladuthurai district (part of Nagapattinam district till 2020) in the southern Indian state of Tamil Nadu. The panchayat is located at the tail end of the Cauvery river on the Coromandel coast, around 2–3 km from the seashore adjoining the Bay of Bengal. The district was among the worst-affected places in the world during the 2004 Tsunami and has experienced four cyclones and multiple episodes of extreme rain or drought between 2008 and 2019 (Government of Tamil Nadu, 2021). Recurring high tides, excess seawater intrusion and increasing sea levels have affected the river outlets and groundwater, increasing their salinity and have also increased the risks of flooding and salinisation. Furthermore, in the absence of any safety measures, seasonal sea breeze often dries up the crops.

The village's soil ranges from slightly saline to calcareous (or chalky) coastal alluvial and is characterised by slow infiltration and moderate runoff. The amount of dissolved salts in the soil (called soil electrical conductivity (EC)) ranges from 3–4.98 millimhos/cm. This means that while the soil is only suitable for salinity-tolerant crops like cotton, it is not suitable to grow paddy, the main food crop (which has a threshold limit of 3 millimhos/cm), using ground water, which is also increasingly saline (discussed below, although we have not measured the EC of water).^3^ The soil also has high iron content and thus high toxicity, also a sign of high salt-level in the soil. In Sirkali block as a whole, just under 60% of total area is affected by salt water ingression and the shallow and medium aquifers have poor quality water while water in the deeper aquifers has better quality of water (Central Water Commission, 2017).

The panchayat has a population of 6853 with 2148 households spread over seven hamlets (Table 1). We have only studied the five hamlets which are primarily dependent on agriculture. Of the other two, one predominantly inhabits the fishing community and the second has abandoned crop farming almost entirely in favour of shrimp aquaculture.

**Table 1.** Demography and livelihood profile of different hamlet in the panchayat (2019).

Hamlets	Community^a^	Population	Household	Main livelihood	Main crops cultivated	Livestock (small-scale/backyard)	
*Study hamlets*							
Hamlet 1	SC	156	64	Agricultural wage work, farming in de-facto land and construction work	Paddy	Cow, goat, chicken	
Hamlet 2	SC	288	116	Agricultural wage work, farming in de-facto land and other non-farm work	Paddy	Goat, Chicken, Guinea	
Hamlet 3	MBC, SC	207	78	Farming in own land, agricultural wage work, fish unloading labourers and construction work	Paddy	Cow, buffalo, Chicken	
Hamlet 4	MBC, BC	1683	466	Agricultural wage work, farming in own land and micro enterprises	Paddy, Vegetables	Cow, goat, chicken	
Hamlet 5	MBC, BC	395	154	Farming in own land and agricultural wage work, brick work	Paddy, cotton, ground nut, black gram/green gram, Mango, coconut	Cow, goat, chicken and exotic birds	
*Other hamlets in the village*							
Hamlet 6	MBC, OC	935	320	Prawn cultivation, agricultural wage work	-	Goat, Cow	
Hamlet 7	MBC (Fishermen)	3189	950	Fishing	-	Goat	

a Backward Classes (BC), Most Backward Classes (MBC), Scheduled Castes (SC), Open Category (OC) as determined by the government.

Source: Block Development Office and Village Administrative Office, CVP.

The data collection happened between September 2019 and March 2020 through multiple rounds of fieldwork. A baseline survey was combined with different qualitative methods such as focussed group discussions (FGD), semi-structured interviews, participatory social and resource mapping and transect walk. The FGDs were held separately with male and female farmers. Each FGD group included 8–10 respondents and sampling was done to ensure a balanced representation of households across all castes and classes in the panchayat. The participatory mapping exercises allowed us to obtain a birds’ eye view of the village and its key issues, while semi-structured interviews were used to explore specific issues with more depth. Additionally, we obtained secondary data on land use patterns and area under agriculture from different sources, especially the village administrative office.

Table 1 shows that the castes recognised as the Most Backward Classes (MBC) constitute a majority in this panchayat and are the dominant caste. While historically most MBC households owned land but over time, many have now become landless. The landless MBCs work in construction (both locally and through migration, in urban areas), livestock-rearing and to some extent, farm wage labour and as labour for fishing communities in neighbouring hamlets. However, farm wage labour is mainly done by Scheduled Castes (SC) or Dalits, most of whom are landless. As in the rest of India, Dalit households face severe social and economic discrimination. For example, they reside in isolated settlements away from the main hamlet and face considerable difficulties in trying to lease-in land.

As per our baseline survey in the five study hamlets, there are 246 marginal landholders (up to 1 ha) (most are MBC), 18 small landholders (1 to 2 ha) and 3 medium landholders (2 to 10 ha) involved in the cultivation process and none from the study area have large landholding (over 10 ha).^4^ Landowning households may or may not have both wetland (low-lying area receiving canal water for irrigation) and dryland (upland, with no access to irrigation).

## A confluence of factors

Like most coastal areas, CVP is also naturally prone to salinisation. The district where CVP is located has been identified as highly vulnerable to coastal flooding and erosion (Central Water Commission, 2017; Rajan et al., 2019). However, a range of factors within and beyond the village have led to soil salinisation. While the empirical focus of our fieldwork has been on the village, here we combine primary data and secondary literature to develop a cross-scalar view of the drivers of soil salinity in CVP.

Firstly, the geographical location of the village at the tail end of Cauvery river i.e. in the upper catchment has meant that fresh water scarcity for irrigation has been a problem here historically. This is important since less fresh water flow increases the salinity of the groundwater. The Mettur dam, constructed in 1934, on the Cauvery river brought freshwater irrigation to CVP and surrounding villages through the canals and remained the only reliable source of irrigation for decades. However, in recent years, a combination of inadequate rainfall and political conflict^5^ over the Cauvery river has led to inadequate water supply. The latter in particular leads to otherwise avoidable delays in the release of water from the Mettur dam.

To be sure, there could be other sources of freshwater. Though there are around fifteen ponds in this panchayat, only three are currently in use for agriculture and at the time of research, there were hardly any efforts being undertaken or planned by the government to maintain them.

In addition to the growing shortage of fresh water for irrigation, a major blow came in the form of sand mining in the 1990s in the drainage channels, river bed and lands where groundnut and dryland crops were cultivated. While the problem of fresh water is one generated both due to geographical, environmental and political factors in the region, sand mining was driven by the process of urban growth around CVP. People within and around the village engaged in sand mining for construction of houses and other real estate in and around the village and the neighbouring town. However, it was done illegally in the name of dredging with the complicity of the contractor who was desilting the riverbank. The extent of sand mining has now declined considerably but it allowed seawater to intrude into and stagnate in the mined pits during high tides and tidal surges. This stagnant saline seawater has contaminated not just the groundwater but also fertile soils in the farmlands around it.

Arguably, the most significant cause of soil salinisation in the village in recent decades is the steady growth of unregulated shrimp farms. In 1992, a private company bought more than 40 ha of farmland for shrimp aquaculture. It was bought by another company after a few years which leased out the shrimp farms to local farmers through a bidding process. These farmers were local MBC households who had some surplus to invest and benefited from it. But shrimp aquaculture had led to an increase in groundwater salinity and increased groundwater extraction, which is used to breed the shrimps, and was beginning to lead to water shortages for crop cultivation and domestic use. Thus, soon after, the company was pressured by other locals to stop production.

However, as the resistance ebbed, some farmers who had experience of shrimp farming earlier and the capital to invest in it re-started it after 2000. This time, some other farmers followed suit as well either because they too had the capital or because their lands were ruined for crop production due to increased soil salinity in any case. At the time of our research, such shrimp farms covered more than 57 ha of agricultural land (Table 2) and a total of 54 farmers were engaged in this with the numbers said to be increasing continuously (in this sense shrimp aquaculture is both a cause and an effect of land degradation). However, these farmers did not get approved licenses from the Marine Products Export Development Authority (MPEDA) and were not following any safety regulations necessary to prevent water and soil contamination. These unregulated shrimp farms stored brackish water in shrimp farms without sealing the bottom. They also used excessive amounts of antibiotics, nutritious supplements, reared more shrimp than the prescribed quantity and had poor water treatment practices. Not only did they let out the untreated wastewater from the farms to the common streams and canals, they dumped the waste along with the chemicals into the Cauvery river during the annual farm maintenance. The concerned government authorities have turned a blind eye to these irregularities. In the words of one respondent, ‘We can't tell them to stop shrimp farming as it has become commercialized… Outsiders cannot even bear the stinky smell from shrimp farms that we have to live with.’

**Table 2.** Land utilisation in CVP in 2019-20.

				Land utilisation					
				Land in the name of private company (ha)^a^					
Land classification	Area in ha	Area under cultivation (ha)	Small scale Prawn Farms (ha)	Cultivated	Uncultivated/barren	Total	Current fallow land (ha)	Barren land (ha)	Land not available for cultivation (ha)
Wetland	420.2	183.6	57.08	60.7	107.9	168.6	6.8	4.15	0
Dryland	221.4	27.98	0	0	32.8	32.8	34.8	6	119.82
Uncultivated land	9.1	NA	0	0	0	0	0	0	NA
Porampokku land/ unregistered land	145.1	0	0	0	0	0	0	0	0
Fallow	5	NA	0	0	0	0	NA	0	NA
Total	800.8	211.58	57.08	60.7	140.7	201.4	41.6	10.15	119.82

Source: Agriculture Office, CVP.

a Note: Minor errors in government records in this category were edited after validation by the research team during fieldwork.

Shrimp farms also adversely impact the availability of groundwater for agriculture as they extract indiscriminately. This in turn paves the way for lateral seawater intrusion^6^ making the groundwater more saline, unsuitable for crop production and domestic use. According to the respondents, they could pump out groundwater from the depth of 30–40 feet across all seasons till around 2000. However, this is no longer possible and at certain places in the village, the depth of the groundwater is almost 100 feet below surface level.

It is important to note that the growth of shrimp aquaculture is not a localised development but a manifestation of global and national policy push towards it and of business interests in shrimp. As in the rest of India, shrimp aquaculture first boomed in Tamil Nadu in the 1990s, declining during the 2000s due to diseases affecting yields, overuse of chemicals and the destruction caused by the 2004 Tsunami. It subsequently picked up in the 2010s after the introduction of a Pacific Ocean shrimp variety called *Litopenaeus vannamei* or the whiteleg shrimp. Over the years, the Indian government has encouraged shrimp aquaculture as a strategy for economic development in coastal areas and to promote the country's marine-based exports, leading to India becoming the second largest shrimp producer in the world (Govindarajan, 2017, Salunke et al., 2020), although Tamil Nadu has a small share in this (Govardan, 2020). Studies also show that entities interested in expanding shrimp aquaculture in India could range from large corporations to smaller scale traders/industrialists and village-level capitalists (see Adduci, 2020). Globally too, shrimp aquaculture is promoted as a major strategy for climate change adaptation in coastal areas with growing soil salinity (Paprocki and Huq, 2018). CVP's articulation with national and global political economies in this way thus must be seen as integral to the process of land degradation locally.

Finally, a jetty constructed by the government on the coast of the village in the aftermath of the 2004 tsunami adversely impacted the farmers. In a clear example of the conflict of interests involved in managing land, it was constructed to help the fishermen to move their boats freely into the sea. But its construction disturbed a natural barrier that protected seawater intrusion into the canal. The flow of canal water changed throughout the day, with the seawater flowing inwards during the high tide and tidal surges, mixing with canal water and sometimes spilling over into paddy fields as well. This worsened an already severe salinity problem.

**Figure 1.**
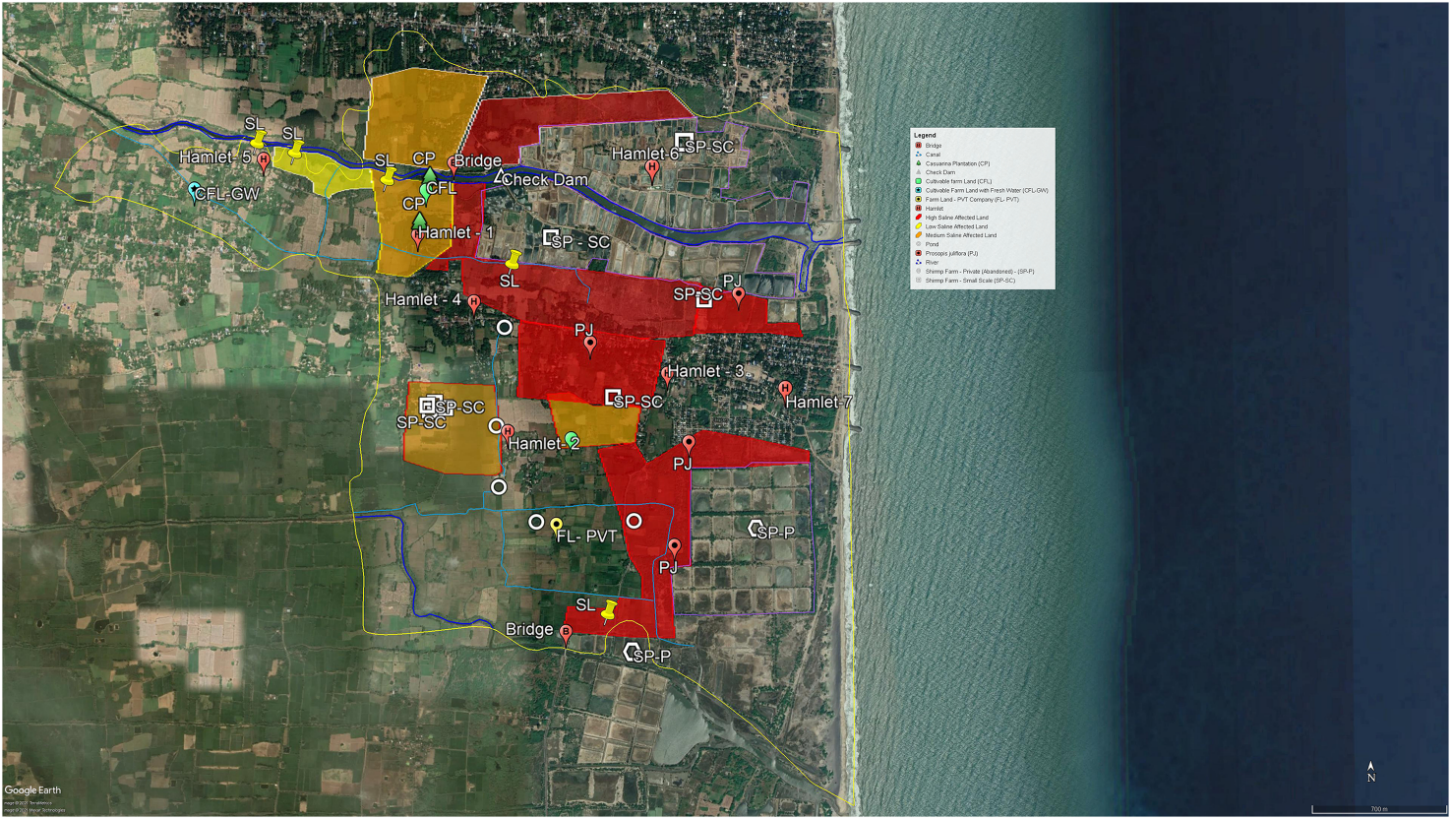
Coastal village panchayat (CVP), Mayiladuthurai district, Tamil Nadu, India.

The growing soil salinity in the village as a result of the above is represented spatially in Figure 1. Areas marked in red are those of high salinity while those marked in orange have medium salinity. The eastern part of hamlets 3 and 4 are more affected by salinity and the MBC landowners here have completely stopped agriculture. As is evident from the map, the western part of the village panchayat is still relatively secure from destructive levels of salinity. Most of the land in hamlet 5 is also still under crop cultivation. A check dam constructed recently, in 2020, will prevent the entry of saline water into the river and potentially revive the agriculture near the river bank area and hamlet 5 farm lands should be saved from the high levels of salinity. But even this will not prevent or address the salinity issue in the lands south of the river, which forms the greater part of CVP. The map also shows that the areas adjacent to the greater majority of shrimp farms are highly saline and occupied by *Prosopis juliflora*, an invasive species (see Section V), and are thus uncultivable. Meanwhile, cultivable farm lands near a single or a smaller number of shrimp farms, that are still fit for cultivation in at least one season, could easily become completely uncultivable in a few years unless preventive measure are introduced.

As this section has shown, a mix of local and extra-local factors over three decades have resulted in severe land degradation. The geographical conditions of the village at the tail end of the river has combined with the shift towards shrimp aquaculture by the better-off households, political issues such as the conflict over the Cauvery river, illicit sand mining and the ill-planned jetty to result in severe soil salinisation in CVP. We now turn our attention towards the resulting changes in land use patterns in the village.

## Changes in land use and cropping

The growing soil salinity in the village has led to major changes in land use and cropping patterns in CVP. Table 3 shows the changes in the area under cultivation between 1991 and 2011 while Table 2 shows the pattern of land utilisation at the time of fieldwork in 2019–2020. Table 3 clearly shows that both irrigated and rainfed area in the village declined between 1991 and 2011 with the total area under cultivation declining from 440.08 ha to 284.1 ha, i.e. a fall of around 35%. While the data in Tables 2 and 3 may not be strictly comparable as they were gathered by different government bodies, it is notable that the total area under cultivation in 2019–20 was even lower at 211.58 ha.

**Table 3.** Changes in area under cultivation of CVP.

Year	Area under Irrigation (ha)	Area under Rainfed (ha)	Total area under cultivation (ha)
1991	387.7	52.38	440.08
2001	255.6	18.5	274.1
2011	274.6	9.5	284.1

Source: Part XII A - District Census Handbook, Census of India for the years 1991, 2001 and 2011.

Table 2 shows that at the time of fieldwork as much as 28% of total wetland was either uncultivated or barren, while around 13% was under shrimp cultivation. Out of the total wetland 420.2 ha, farmers were able to cultivate only 244.3 ha (183.6 ha of own land and 60.7 of company land, explained next) or around 58% of the wetland in 2019, and the remaining areas became uncultivable and are utilised for other purposes including shrimp aquaculture. The purchase of wetland by private companies (168.6 ha) considerably reduced the utilisation of land for agriculture. However, as we explain below, none of it is in use by the companies and local farmers have negotiated informally with one of them to use 60.7 ha for paddy cultivation.

The gradual but continuous land degradation described here has also led to drastic changes in the cropping pattern. Table 4 indicates that till the 1980s, a wide variety of crops were being cultivated in the area across all three seasons: *kuruvai* (June-August), *samba* (September-January) and *thaladi* (January-March). Traditional varieties of paddy, vegetables, groundnut and millets had been cultivated depending on the season and, except in the peak summer months of April and May when the land is prepared for the monsoon crop, people could cultivate the land with something throughout the year.

**Table 4.** Trend analysis- changes in cropping pattern for 40 years.

Year	Season		
	Kuruvai (Kharif- June to Aug)	Samba (Rabi-Sept to Jan)	Thaladi (Rice fallow- Jan to Mar)
Till 1980	Traditional paddy variety	Traditional paddy variety	Paddy, black gram and green gram, groundnut & millets (pearl millet, finger millet & sorghum) and vegetables
1980-2000	High yield paddy varieties	High yield paddy varieties	Paddy, black gram and green gram, maize and vegetables
After 2000	No cultivation- Fallow land (except in Eramplayam- high yield varities)	High yield paddy varieties	Black gram and green gram, maize; cotton (where borewell facility available)

Source: Own fieldwork, 2019-20.

With the spread of Green Revolution technologies in the area from the 1980s, there was a shift towards high-yielding varieties of paddy. However, in the decades since 2000, there has been a drastic change towards land being left fallow in the kuruvai season. For many decades now, cultivation in the kuruvai season has happened using water from the Cauvery river and groundwater, and both sources have become more saline, leaving farmers with little choice but to leave the land fallow. To make matters worse, where land has been left fallow for more than two seasons, the invasive tree *Prosopis juliflora* has grown. Leading to further depletion of groundwater and deterioration of land quality, this species is highly difficult to uproot, making it almost impossible to restore the land if left unattended.

**Figure 2.**
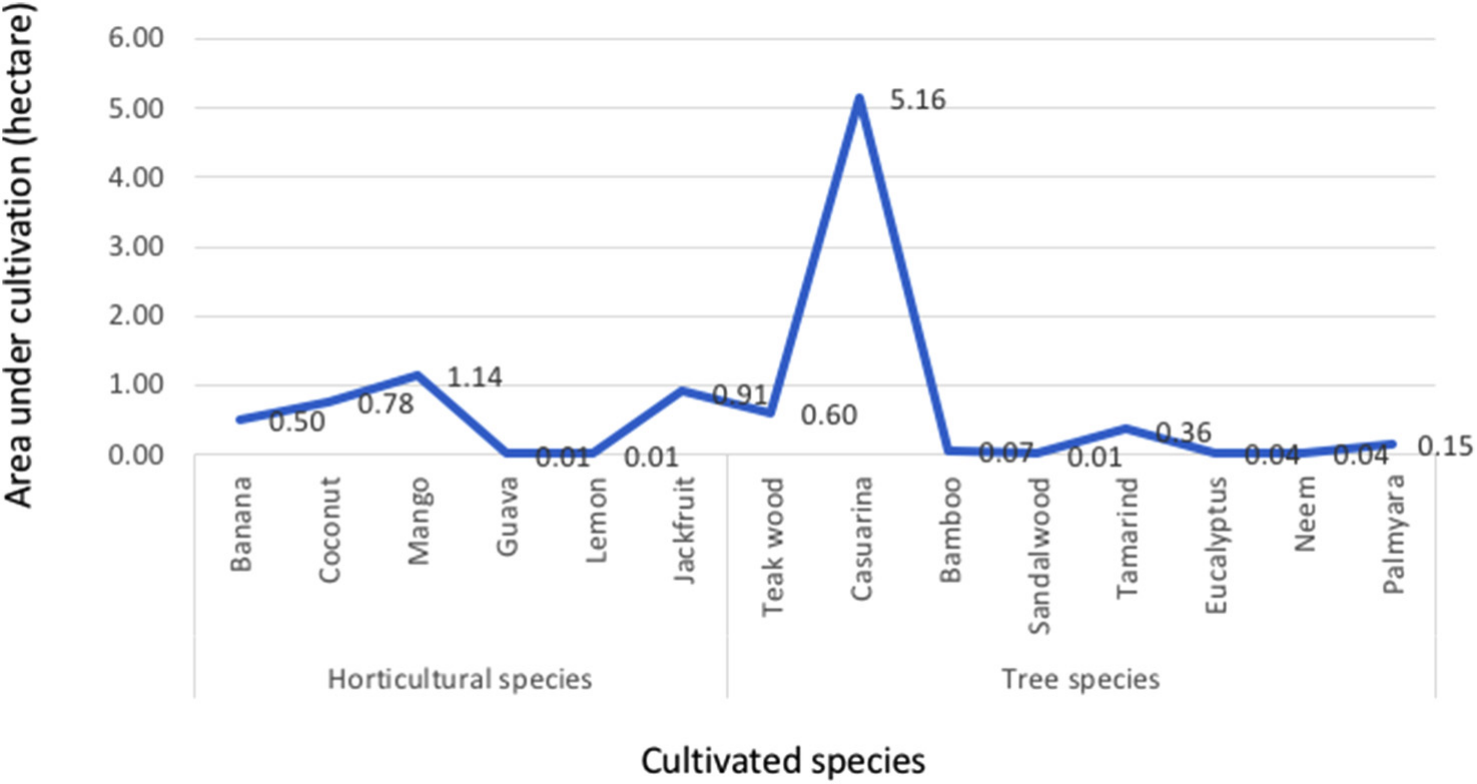
Area under horticultural and arboreal species in 2019.

Thaladi is the third crop of the year, when relatively less paddy is cultivated and is thus called ‘rice fallow’. Based on the water availability in the canal, farmers would intermittently grow paddy or groundnut and vegetables. However, the soil has now become too saline to cultivate any of these crops. There has also been a reduction in the horticultural and arboreal species cultivated in CVP since the 2000s (Figure 2). The mango orchards and coconut trees grown by some farmers on their drylands are dead and farmers cannot grow any horticultural crops on these lands. Some farmers have resorted to cultivating casuarina in the dryland but at the time of fieldwork this amounted to a total of only around 5 ha.

In the wetlands, farmers started cultivating cotton in the thaladi season, using water from borewells, in order to cope with soil salinity. But even this was possible only where the salinity was mild to moderate: cotton does not grow well in conditions of high salinity nor do borewells function well. Not only does this add further strains on groundwater, the shift to cotton cultivation (42.92 ha; see Table 5) is also significant in other ways. Women control paddy production in these villages and the shift to cotton implies loss of their control over production. Moreover, unlike paddy where the farmers receive the government-mandated Minimum Support Price, cotton prices can be volatile, contributing to household income insecurity. A female farmer stated unequivocally, ‘Salinity is the main reason for changing our cropping pattern. We never planted cotton before; only after the salinity in our farmlands grew, we took it up. We used to cultivate paddy in all three seasons till two decades ago but now we cultivate paddy only in samba season.’

**Table 5.** Area under crop cultivation in different season in 2018-19.

Crop	Kuruvai	Samba	Thaladi
	Area of cultivation (ha)		
Paddy	5.93	209.1	-
Cotton	-	-	42.92

Source: Village Administrative Office, CVP.

The samba season (September-January) is the only promising cropping season for the farmers as the area receives water from Cauvery only around September and the north-east monsoon, the sowing period for samba. In the other two seasons (kuruvai and thaladi), less freshwater flow aggravates the soil salinity level. Table 5 shows the distribution of the major crops – rice and cotton – across the three seasons in CVP in 2018-19.

The changes in land use and cropping pattern described here demonstrate the threat of food security and livelihoods as a result of increasing soil salinity. The accompanying shifts towards cotton production, extraction of groundwater using borewells and expansion of shrimp aquaculture are likely to further damage the land. We now turn to how the impact manifests for different social groups.

## Differentiated impacts

In this section, we demonstrate that the impact of growing soil salinity has been differentiated along the axes of class, caste and gender and that the forms that these impacts have taken exacerbate both environmental issues and social inequalities. We remind the readers that there are some overlaps between caste and class in terms of land ownership, ability to lease-in land and types of labour (Section III).^7^ Therefore, the discussion on caste has been merged with that on land and labour, but we signpost specific ways in which caste becomes salient in understanding these changes.

### Land and caste relations

Firstly, there is a clear caste-class divide between those who could adopt shrimp aquaculture and those who could not. We noted earlier that an overwhelming majority of landowners in the five hamlets are marginal landholders and most of them are MBC, and that it was the relatively well-off among them who started shrimp aquaculture. As paddy cultivation is economically unviable when the soil is saline, a third of all landowners had shifted to shrimp farming at the time of our research: some among them had moved out of agriculture completely while some had continued to a limited extent with crop production as well. So, there is not always a neat division between shrimp producers and others.

However, not all landowners could make even a partial transition to shrimp aquaculture, and it is telling that there are no landed Dalits who have shrimp farms. Setting up shrimp farms requires substantial capital investments which was out of reach for many households. But as discussed above, when a paddy field is converted to a shrimp farm, it enables saline water intrusion which in turn triggers a cascading effect on land use in the adjoining fields too. Thus, conditions of crop cultivation by other households also deteriorated, leading to unstable yields and declining returns. Thus, many landowners have been forced to either limit themselves to cultivating only one crop a year or to leave the land fallow. Even if such households wanted to resume crop cultivation, it is almost impossible for them to do this as their abandoned paddy fields have been occupied by the invasive *Prosopis juliflora* trees. Those who left the land fallow are also unable to keep livestock, a crucial source of household income, because of lack of access to fodder. This has important gendered implications, discussed below.

It is worth noting the differential returns from shrimp vis-à-vis paddy here. Although we did not calculate detailed estimates of the profits and loss of the shrimp cultivation, in multiple FGDs, respondents noted that most shrimp farmers made a profit of around 60–70% (unless there is a disease or disaster) while paddy farmers made a profit of 10% or less. This shows that land degradation was not a problem but an opportunity for the minority of landowners that succeeded in transforming into shrimp farmers. They have been the winners of this process alongside the private businesses invested in the shrimp business through credit, input or export. We have not investigated the political economy of shrimp aquaculture in this region in detail. Nevertheless, the basic point that ‘one person's degradation is another's accumulation’ (Blaikie and Brookfield, 1987: 14) holds. The tension between those cultivating shrimps and those who are not is well-represented in this statement by a farm from Hamlet 4 (see Figure 1, centre-west): ‘we didn't allow the shrimp farms to emerge on one side of the water channel. But the other side of the water channel is full of these farms. Rich persons in the village are doing it as a business. As we are poor, we can't raise our voice against them and even the government only supports them.’

The vulnerability of those who could not shift to shrimp aquaculture is also highlighted by other parallel developments in the village. In 2008, some of the landed MBC and landed Dalit households sold their salinity-affected farmlands to a private company. The latter promised to start a castor oil factory on the land, where these landowners would be given jobs. Many were in desperate need of money and sold the land at the relatively low rate being offered (Rs. 40,000/ha). Some others negotiated the price and managed to get a higher rate of Rs. 240,000/ha. In any case, the company fenced off the land but did not set up the factory, instead using it as a mortgage to secure a bank loan. After waiting in vain for a factory job for a few years and with no other secured livelihood at hand, the households broke the fence, made an informal agreement with the company and started cultivating their respective plots, only this time without a title deed. This has made them even more vulnerable than they were before.

As more and more cultivable land is becoming unproductive due to salinity, the possibilities of leasing in land for cultivation by both the landless and landed households have also declined. Landless Dalits always found it difficult to lease in land. However, this has now become a wider problem. Land is leased out not only by those who are disillusioned from agriculture or simply do not have household labour to be involved in agriculture, but also those who are in desperate need of cash. At the time of fieldwork, the most common system was that the lessor leased out an acre or 0.4 ha of land without borewell at Rs. 20,000 (allowing cultivation of paddy) and the same amount of land with borewell at Rs. 50,000 (allowing cultivation of paddy and cotton) for three years. This amount, paid by the lessee to the lessor at the start of the three years, needed to be returned to the lessee at the end of the three years. It effectively worked like a cash loan without interest to the lessor. The growing unproductivity of land has, therefore, also undermined this strategy to alleviate distress. The exception is Hamlet 5. Since it is away from the shrimp farms (see Figure 1), it is less affected by salinity and land leasing is said to have increased here in recent years.

### Labour and migration

The reductions in cropping intensity and area under crop cultivation have led to a significant decline in farm wage labour. Only about a month of agricultural work is available locally while shrimp aquaculture is not as labour-intensive as paddy. While labour on shrimp farms is hired from the village, most are hired for night security. As these are small-scale farms, all the other work is usually done by the farmers’ themselves or by family labour.

As landless Dalits constitute the largest group of agricultural labourers, they are most affected by these developments as a social group. But to the extent that some landless and marginal MBC households also did farm wage labour, they too are affected. Consequently, men across households in the study area, up to the age of around forty-five years but especially young men, have shifted to non-agricultural work. Both young men and their parents are disillusioned by land-based livelihoods due to environmental and economic risks and aspire for them to earn an income through other sources. But government jobs are scarce and working as migrant labour in the precarious informal sector is usually their only option.

However, here too, there is a difference in the kind of work done by different groups. A large majority of landed and landless MBC men migrate to cities or other states to work as masons or in the carving and painting of Hindu temples. While this is not their traditional caste-based occupation, they have acquired these skills over time through contact with an MBC group specialising in temple design that migrated to the village from Myanmar in the 1960s. Dalit men, on the other hand, are far less likely to be doing this kind of work. Dalit men from one hamlet have learnt the skill of temple carving and painting but they are an exception. Most work as helpers to masons or in the more exploitative brick-making industry. The study area also has overseas migrants, i.e. male members who have migrated to Singapore or Dubai, but they are almost exclusively from landed MBC households. The majority of the landed male migrants return to the village during paddy cultivation during the samba season to attend to their farmlands, while the landless migrants and those who are over fifty years of age are less likely to return.

To be sure, migrant households, whether domestic or international, are often considered more economically secure compared to the non-migrant households of the same community. Non-farm incomes were regular and assured and the daily wages were almost double of the daily wages in agriculture. For example, in 2019, while the average daily wage for men in agriculture was Rs 550, as a mason and a helper to a mason it was Rs 800 and 600, respectively. However, this is casualized, informal work which is known to be highly exploitative in India (see Kannan, 2018). Moreover, the degradation of agricultural land also represents loss of an important asset for subsistence needs and has gendered implications, to which we turn to next.

### Women's work

Though the decline of paddy cultivation displaced agricultural work for both men and women, paddy cultivation employed more women than men. Women across landowning and caste groups were also traditionally completely reliant on small-scale livestock as a regular source of income through the sale of milk and as an asset that could be sold to obtain lump sum cash for their children's education or marriage. As soil salinity has affected fodder availability as well, such livestock-rearing has been undermined. Meanwhile, women are not involved in any aspect of shrimp aquaculture. Thus, women's income-earning capacity has been disproportionately impacted by the land use changes described here. This has serious implications on household food and nutrition security. In our FGDs and interviews, women reported that the wages of men are spent on their personal expenses and not towards overall household needs. Moreover, groundwater salinisation has also led to a severe deterioration of water for domestic and consumption purposes, leading to women spending more time and energy to arrange potable water.

While men from MBC households were migrating for non-agricultural work to cities, women of MBC households were increasingly taking the responsibility of agricultural production and marketing. Earlier too, these women were involved in transplantation, weeding, and harvesting the paddy straw. However, they now shouldered the responsibility of managing the whole cultivation process, including labour-hiring. At the same time, it should be noted that in relatively better-off households, even when the men migrate, the women do not do such work. On the other hand, Dalit women do agricultural work on leased in (or sold) land and/or as labour on others’ lands without exception.

Not just caste and economic position but age and marital status also play a role in women's participation in agriculture. Dalit women do agricultural work regardless of their age and marital status. But MBC women from landless households are allowed to work only after their marriage with their husband's consent. Landed MBC women, on the other hand, usually only do managerial work in agriculture even after marriage. Where the husbands in these landed MBC households have migrated, the women see their involvement in agriculture only as a way of earning some additional income.

Dalit women, women from landless, women-headed or otherwise poor MBC households were also found to work as helpers to masons. Not only are the wages higher in the latter (Rs 250 per day compared to Rs 150 a day in agriculture), employment is also available throughout the year (as against seasonally in agriculture). Those women who could not shift to non-agricultural employment owing to caring responsibilities at home engaged in very small-scale businesses like selling *idli* (steamed rice cake) batter and/or yoghurt, running petty grocery shops or selling firewood in order to generate some regular income for the household.

The data presented here shows that currently, many women are intensifying and diversifying their work towards the non-farm sector within their village/habitation as a coping measure. In negotiating these changes, they have inadequate access to finance from formal financial institutions. Some of these women were part of a Self-Help Group in the past but this is defunct now. They are thus either dependent on their own savings or on exploitative informal sources for their small credit needs. Such women also depend on the Mahatma Gandhi National Rural Employment Guarantee Scheme (MGNREGS) which assures 100 days employment in a year per person. However, right now they are getting only 20–25 days of work in a year.

This analysis of differentiated impacts shows that land degradation is worsening existing inequalities. In this sense it manifests what Rigg et al. (2016) have described as the difference between inherited and produced vulnerability. The inherited vulnerabilities of land-based, caste and gender inequalities have been intensified by shrimp aquaculture by better-off MBC households which makes any land-based livelihood increasingly unviable. However, by framing these vulnerabilities in terms of inequalities, we also underline that the way vulnerability is ‘re-worked’ (Rigg et al., 2016, 66) is also relational.

## Conclusion

This paper focused on degradation of agricultural land in a coastal delta region, thereby filling an important gap in the literature on coastal degradation. With a focus on soil salinity, it argued that the biophysical degeneration of land is not just caused by a combination of human and environmental factors but also that it generates social contradictions that further intensify both land degradation and social inequalities. In our case study in south India, it has been driven by a complex interaction between environmental vulnerabilities, forces of commoditization, political conflicts and existing social hierarchies over a long period of time – here, we have traced the process of degradation from the 1990s onwards over a period of three decades. We have demonstrated that through such a configuration of the process of land degradation, some social actors are driving, even benefitting from, degradation while others are disproportionately adversely impacted by it, and this has worsened existing social hierarchies. Thus, far from being passive recipients of an external process of degradation, the way in which the socially differentiated local population has responded to the land degradation has in fact structured and intensified the process of degradation itself.

A small section has been incentivized to engage in shrimp aquaculture while the agricultural livelihoods of the vast majority are increasingly strained. While Dalit households have always been worse off, there is increasing distress even among a section of MBC households due to growing salinity in their farm lands and thus declining farm income. Male outmigration, especially of the youth, also intersects with caste and land ownership (class), and more often than not adds to the work burdens of women. The continuous shift away from paddy also has implications for household food security. Meanwhile, on the one hand, unregulated shrimp aquaculture is increasing soil salinity and contamination and, on the other, the abandonment of agricultural land due to its unproductivity is leading to a growth of invasive species.

There is an almost vicious cycle whereby there is either no interest or no capacity locally for their agricultural lands to be restored or at least for further salinisation to be prevented. It is telling that the government's disaster management plan for the Mayiladuthirai district (Government of Tamil Nadu, 2021) does not engage with any specific causes or mitigation strategies associated with the overwhelming issue of soil salinity. Ultimately, restoration of the land requires concerted political action by the government at various levels. For instance, the provisions of the MGNREGS allow it to be used for land restoration (Ministry of Rural Development, 2020). A range of activities, such as check dams, deepening of canals on a regular basis, bund plantation and renovating traditional water bodies, can both control salinity and create a hundred days of employment within the village for men and women. While this alone may not be a comprehensive solution, it would go a long way to alleviate some of the distress experienced by a large majority of the local population. However, the government has made no efforts towards this.

Recently, the Tamil Nadu government approved the Protected Agricultural Zone Development Act which seeks to restrict industries on agricultural land within the zone in which the study area falls. Households in our study area who sold their land to the company that promised to develop a factory are now trying to regain formal control of their land. However, here again, Ramesh and Babu (2020) have argued that the Act ‘only regurgitates old solutions and proposes no actionable steps’. The state's inability, or perhaps unwillingness, to recognise coastal degradation as a problem of structural inequality not only prevents any remedies, it aids the downward spiral.

Such inadequacies, of course, are not a problem for everyone. They work to the advantage of those who no longer depend on paddy or other crop cultivation, such as small-scale shrimp farmers, and other economic actors interested in agricultural land for non-agricultural purposes. So as public will and execution continue to falter, studies such as this one that bring together environment, society and policy become more urgent than ever to reveal the dynamic and deeply contentious nature of land use and degradation and to explore possibilities for less environmentally damaging and more socially just ways forward.

## Highlights

1. Describes the process of coastal land degradation and its socio-economic impacts over three decades in south India
2. A political ecology and political economy analysis of how land degradation generates contradictions that worsen degradation and social inequalities
3. Draws on intensive qualitative field research using a variety of methods
4. Centres agriculture in the analysis of coastal land degradation

## Notes

1. This is inspired from Baglioni and Campling’s (2017) articulation of the dialectical relation between nature and society in the context of the natural resource-based global value chains.

2. In parts of Tamil Nadu, excessive withdrawal of groundwater has been reported as a primary triggering factor for seawater intrusion rather than climate change (Kanagaraj et al., 2018).

3. To be sure, low EC of ground water can balance off the effects of soil salinity on paddy production under favourable soil and climatic conditions (Ayers and Westcot, 1985). Note also that other features of soils such as texture (mainly clay loam), not discussed here, can also affect crop yields under the conditions of increasing both soil and ground water salinity (e.g. Guo et al., 2012).

4. This is the classification used by the Government of India.

5. The Cauvery water dispute is a legal dispute about water-sharing between the southern Indian states of Tamil Nadu and Karnataka which dates back to the late 19^th^ century (Mantri, 2018).

6. When groundwater is pumped from a coastal aquifer, lowered water levels can cause seawater to be drawn toward the freshwater zones of the aquifer. This decreases the freshwater storage in the aquifers. Without treatment, this groundwater does not conform to drinking-water or agricultural water-quality standards.

7. This is the norm across India (Lerche and Shah, 2018).
